# Analysis of codon usage and nucleotide composition bias in polioviruses

**DOI:** 10.1186/1743-422X-8-146

**Published:** 2011-03-30

**Authors:** Jie Zhang, Meng Wang, Wen-qian Liu, Jian-hua Zhou, Hao-tai Chen, Li-na Ma, Yao-zhong Ding, Yuan-xing Gu, Yong-sheng Liu

**Affiliations:** 1 State Key Laboratory of Veterinary Etiological Biology, Lanzhou Veterinary Research Institute, Chinese Academy of Agricultural Sciences, Lanzhou, 730046 Gansu, China

## Abstract

**Background:**

Poliovirus, the causative agent of poliomyelitis, is a human enterovirus and a member of the family of Picornaviridae and among the most rapidly evolving viruses known. Analysis of codon usage can reveal much about the molecular evolution of the viruses. However, little information about synonymous codon usage pattern of polioviruses genome has been acquired to date.

**Methods:**

The relative synonymous codon usage (RSCU) values, effective number of codon (ENC) values, nucleotide contents and dinucleotides were investigated and a comparative analysis of codon usage pattern for open reading frames (ORFs) among 48 polioviruses isolates including 31 of genotype 1, 13 of genotype 2 and 4 of genotype 3.

**Results:**

The result shows that the overall extent of codon usage bias in poliovirus samples is low (mean ENC = 53.754 > 40). The general correlation between base composition and codon usage bias suggests that mutational pressure rather than natural selection is the main factor that determines the codon usage bias in those polioviruses. Depending on the RSCU data, it was found that there was a significant variation in bias of codon usage among three genotypes. Geographic factor also has some effect on the codon usage pattern (exists in the genotype-1 of polioviruses). No significant effect in gene length or vaccine derived polioviruses (DVPVs), wild viruses and live attenuated virus was observed on the variations of synonymous codon usage in the virus genes. The relative abundance of dinucleotide (CpG) in the ORFs of polioviruses are far below expected values especially in DVPVs and attenuated virus of polioviruses genotype 1.

**Conclusion:**

The information from this study may not only have theoretical value in understanding poliovirus evolution, especially for DVPVs genotype 1, but also have potential value for the development of poliovirus vaccines.

## Background

When molecular sequence data started to be accumulated nearly 20 years ago, it was noted that synonymous codons are not used equally in different genomes, even in different genes of the same genome[1-3]. As an important evolutionary phenomenon, it is well known that synonymous codon usage bias exists in a wide range of biological systems from prokaryotes to eukaryotes [4,5]. Codon usage analysis has been applied to prokaryote and eukaryote, such as *Escherichia coli*, *Bacillus subtilis*, *Saccharomyces cerevisiae*, C*aenorhabditis elegans *and human beings [6-8]. These observed patterns in synonymous codon usage varied among genes within a genome, and among genomes. The codon usage is attributable to the equilibrium between natural selection and mutation pressure [9,10]. Recent studies of viral codon usage has shown that mutation bias may be a more important factor than natural selection in determining codon usage bias of some viruses, such as *Picornaviridae*, *Pestivirus*, plant viruses, and vertebrate DNA viruses [9,11-13]. Meanwhile, recent report also showed that the G+C compositional constraint is the main factor that determines the codon usage bias in iridovirus genomes[11,14]. Analysis of codon usage can reveal much about the molecular evolution or individual genes of the viruses.

Polioviruses belong to the family *Picornaviridae *and are classified as human *enterovirus *C (HEV-C) species in the genus Enterovirus according to the current taxonomy [15,16]. Polioviruses can be divided into three different genotypes: 1, 2 and 3. The genome of each genotypes contains a single positive-stranded RNA with a size of approximately 6 kb consisting of a single large open reading frame (ORF) flanked by 5' and 3' untranslated region [17].

As we known, the Sabin oral poliovaccine (OPV) was among the best known viral vaccines [18]. It has saved the lives and health of innumerable people, in particular children. However, poliovirus is highly genetically variable. OPV viruses may undergo transformation into circulating highly diverged VDPV, exhibiting properties hardly distinguishable from those of wild polioviruses [19]. So far, little information about synonymous codon usage pattern of polioviruses genome has been acquired to date. To our knowledge, this is the first report of the codon usage analysis on polioviruses (including wild strains, attenuated live vaccine strains and VDPV strains). In this study, we analyzed the codon usage data and base composition of 48 available representative complete ORFs of poliovirus to obtain some clues to the features of genetic evolution of the virus.

## Methods

### Sequence data

A total of 48 poliovirus genomes were used in this study (Table 1). The serial number (SN), genotype, length value, isolated region, GenBank accession numbers, and other detail information about these strains were listed in Table 1. All of the sequences were downloaded from NCBI http://www.ncbi.nlm.nih.gov/Genbank/, and 48 poliovirus genomes were selected in the study. The other sequences with >98% sequence identities were excluded.

**Table 1:** The information of 48 polioviruses genomes used in this study

SN	Strain	Gene type	Length^a^	Isolation	Note	**Accession No**.
1	CHN-Henan/91-3	1	6630	China	W Virus^b^	AF111983
2	CHN-Jiangxi/89-1	1	6630	China	W Virus^b^	AF111984
3	P1W/Bar65 (19276)	1	6630	Belarus	DVPV	AY278553
4	HAI01008C2	1	6630	Haiti	DVPV	AF405662
5	HAI01007	1	6630	Haiti	DVPV	AF405666
6	HAI01002	1	6630	Haiti	DVPV	AF405667
7	HAI01001	1	6630	Haiti	DVPV	AF405668
8	HAI00003	1	6630	Haiti	DVPV	AF405669
9	DOR01012	1	6630	Dominica	DVPV	AF405670
10	DOR00041C3	1	6630	Dominica	DVPV	AF405679
11	DOR00028	1	6630	Dominica	DVPV	AF405684
12	99/056-252-14	1	6630	Russia	DVPV	AF462418
13	RUS-1161-96-001	1	6630	Russia	DVPV	AF462419
14	HAI01-13	1	6630	Haiti	DVPV	AF416342
15	TCDCE01-135	1	6630	C Taiwan^c^	DVPV	AF538840
16	TCDC01-113	1	6630	C Taiwan^c^	DVPV	AF538841
17	TCDC01-330	1	6630	C Taiwan^c^	DVPV	AF538842
18	TCDC01-861	1	6630	C Taiwan^c^	DVPV	AF538843
19	Sabin 1	1	6630	USA	Vaccine^d^	AY184219
20	Brunhilde	1	6630	China	W Virus^b^	AY560657
21	USA10784	1	6630	USA	DVPV	EF682356
22	USA10785	1	6630	USA	DVPV	EF682357
23	USA10783	1	6630	USA	DVPV	EF682358
24	USA10786	1	6630	USA	DVPV	EF682359
25	CHN8229-3/GZ/CHN/2004	1	6630	China	DVPV	FJ769381
26	10050	1	6630	China	DVPV	FJ859058
27	10091c	1	6630	China	DVPV	FJ859060
28	10092c	1	6630	China	DVPV	FJ859061
29	10094c	1	6630	China	DVPV	FJ859062
30	10095c	1	6630	China	DVPV	FJ859063
31	10097c	1	6630	China	DVPV	FJ859064
32	EGY88-074	2	6624	Egypt	DVPV	AF448782
33	EGY93-034	2	6624	Egypt	DVPV	AF448783
34	P2S/Mog65-3 (20120)	2	6624	Belarus	DVPV	AY278549
35	P2S/Mog66-4 (21043)	2	6624	Belarus	DVPV	AY278551
36	P2S/Mog65-2 (20077)	2	7439	Belarus	DVPV	AY278552
37	NIE0210766	2	6624	Nigeria	DVPV	DQ890385
38	NIE0110767	2	6624	Nigeria	DVPV	DQ890386
39	USA9810768	2	6624	USA	DVPV	DQ890387
40	PER8310769	2	6624	Peru	DVPV	DQ890388
41	32191	2	6624	Belarus	DVPV	FJ460223
42	32189+AP1	2	6624	Belarus	DVPV	FJ460224
43	31996	2	6624	Belarus	DVPV	FJ460225
44	PV2/Rus	2	6624	Russia	DVPV	FJ517649
45	Sabin 3	3	6621	USA	Vaccine^d^	AY184221
46	33239	3	6621	Belarus	DVPV	FJ460226
47	31974	3	6621	Belarus	DVPV	FJ460227
48	FIN84-60212	3	6621	Finland	DVPV	FJ842158

Note: ^a ^the length values excluding non-coding sequence.

^b ^means wild strain

^c ^stands for China Taiwan

^d ^stands for attenuated live vaccine strain

### The actual and predicted values of the effective number of codon (ENC)

The ENC is used to measure the degree of departure from the equal use of synonymous codons of coding regions of polioviruses. The values of the effective number of codon (ENC) range from 20 to 61. In an extremely biased gene where only one codon is used for each amino acid, this value would be 20; if all codons are used equally, it would be 61; and if the value of ENC is greater than 40, the codon usage bias was regarded as low. The values of ENC were obtained by EMBOSS CHIPS program [20].

Genes, whose codon choice is constrained only by a mutation bias, will lie on or just below the curve of the predicted values. The predicted values of ENC were calculated as

where *s *represents the given (G+C)_3_% value [21].

### The calculation of the relative synonymous codon usage (RSCU)

To investigate the pattern of relative synonymous codon usage (RSCU) without the influence of amino acid composition among all polioviruses samples, the RSCU values of codons in each ORF of polioviruses were calculated according to the formula of previous reports [22,23].

where g*_ij _*is the observed number of the *i*th codon for *j*th amino acid which has *n_i _*type of synonymous codons. The codon with RSCU value more than 1.0 has positive codon usage bias, while the value <1.0 has relative negative codon usage bias. When RSCU value is equal to 1.0, it means that this codon is chosen equally and randomly.

### Relative dinucleotide abundance in polioviruses

Because dinucleotide biases can affect codon bias, the relative abundance of dinucleotides in the coding regions of polioviruse genomes was assessed using the method described by Karlin and Burge [24]. A comparison of actual and expected dinucleotide frequencies of the 16 dinucleotides in coding region of the 48 polioviruses genomes was also undertaken. The odds ratio *ρ*_xy_=ƒ_xy_/ƒ_y_ƒ_x_, where ƒ_x _denotes the frequency of the nucleotide *X*, ƒ_y _denotes the frequency of the nucleotide *Y*, ƒ_y_ƒ_x _the expected frequency of the dinucleotide *XY *and ƒ_xy _the frequency of the dinucleotide *XY*, etc., for each dinucleotide were calculated. As a conservative criterion, for *ρ*_ xy  _> 1.23 (or < 0.78), the *XY *pair is considered to be of over-represented (or under-represented) relative abundance compared with a random association of mononucleotides.

### Statistical analysis

Principal component analysis (PCA) was carried out to analyze the major trend in codon usage pattern in different genomes of polioviruses (excluding non-coding regions). It is a statistical method that performs linear mapping to extract optimal features from an input distribution in the mean squared error sense and can be used by self-organizing neural networks to form unsupervised neural preprocessing modules for classification problems [6]. In order to minimize the effect of amino acid composition on codon usage, each ORF is represented as a 59-dimensional vector. Each dimension corresponds to the RSCU value of one sense codon excluding Met, Trp and three stop codons.

Linear regression analysis was used to find the correlation between codon usage bias and gene length. Correlation analysis is used to identify the relationship between codon usage bias and synonymous codon usage pattern. This analysis is implemented based on the Spearman's rank correlation analysis way.

All statistical analyses were carried out using the statistical analysis software SPSS Version 17.0.

## Results

### The characteristics of synonymous codon usage in polioviruses

In order to investigate the extent of codon usage bias in polioviruses, all RSCU values of different codon in 48 polioviruses strains were calculated. There is only two preferred codons UUG (Leu) and GUG (Val), choosing G at the third position, and most of preferred codons are ended with A (Table 2). Moreover, polioviruses genome is A redundant with A content ranging from 29.739 to 30.826.11, with the mean value of 30.367 and S.D. of 0.234; in contrast, low content of G ranging from 21.723 to 22.401 (mean = 22.118, S.D. of 0.147), suggesting that nucleotide contents influence the patterns of synonymous codon usage (Table 3). The values of ENC among these polioviruses ORFs are similar, which vary from 52.609 to 55.105 with a mean value of 53.754 and S.D. of 0.545. The data showed that the extent of codon preference in polioviruses genes was kept basically stable.

**Table 2:** Synonymous codon usage in the coding region of polioviruses

*AA ^a^*	*Codon*	*RSCU ^b^*	*AA*	*Codon*	RSCU ^b^
Phe	UUU	**1.020**	Gln	CAA	**1.075**
	UUC	0.980		CAG	0.925
					
Leu	UUA	0.914	His	CAU	0.787
	UUG	**1.349**		CAC	**1.213**
	CUU	0.566	Asn	AAU	0.900
	CUC	0.909		AAC	**1.100**
	CUA	1.023	Lys	AAA	**1.050**
	CUG	1.072		AAG	0.95
					
Val	GUU	0.441	Asp	GAU	0.961
	GUC	0.762		GAC	**1.039**
	GUA	0.735	Glu	GAA	**1.057**
	GUG	**1.657**		GAG	0.943
					
Ser ^c^	UCU	0.785	Arg	AGA	**2.868**
	UCC	1.345		AGG	1.471
	UCA	**1.749**		CGU	0.434
	UCG	0.424		CGC	0.577
	AGU	0.920		CGA	0.268
	AGC	0.777		CGG	0.381
					
Pro	CCU	0.800	Cys	UGU	**1.105**
	CCC	0.799		UGC	0.895
	CCA	**1.884**	Tyr	UAU	0.847
	CCG	0.517		UAC	**1.153**
					
Thr	ACU	1.170	Ala	GCU	1.161
	ACC	**1.330**		GCC	0.969
	ACA	1.124		GCA	**1.438**
	ACG	0.376		GCG	0.432
					
Gly	GGU	1.160	Ile	AUU	**1.247**
	GGC	0.757		AUC	1.049
	GGA	**1.175**		AUA	0.705
	GGG	0.909			

Note: The boldface means the preferred codon compare with other synonymous codon.

**Table 3:** Nucleotide contents in ORFs of 48 poliovirus genomes

*No*.	*A*	*G*	*U*	*C*	*A_3_*	*C_3_*	*G_3_*	*U_3_*	*A+U*	*G+C*	*C_3_/G_3_*	ENC
1	0.305	0.220	0.236	0.239	0.275	0.270	0.192	0.263	0.541	0.459	0.462	53.082
2	0.305	0.218	0.236	0.242	0.269	0.275	0.191	0.264	0.540	0.460	0.467	53.749
3	0.300	0.219	0.238	0.242	0.263	0.283	0.190	0.264	0.538	0.462	0.473	53.709
4	0.301	0.222	0.236	0.241	0.269	0.279	0.189	0.263	0.537	0.463	0.468	54.085
5	0.308	0.221	0.233	0.238	0.285	0.268	0.189	0.258	0.541	0.459	0.457	53.936
6	0.300	0.223	0.233	0.244	0.265	0.289	0.192	0.253	0.533	0.467	0.481	53.506
7	0.300	0.223	0.236	0.241	0.267	0.279	0.191	0.263	0.536	0.464	0.470	53.929
8	0.305	0.220	0.233	0.242	0.281	0.283	0.185	0.250	0.538	0.462	0.469	53.506
9	0.301	0.222	0.232	0.244	0.270	0.287	0.189	0.253	0.533	0.467	0.477	53.592
10	0.303	0.220	0.232	0.244	0.275	0.288	0.186	0.252	0.535	0.465	0.474	53.308
11	0.303	0.221	0.234	0.242	0.273	0.283	0.187	0.257	0.537	0.463	0.470	53.389
12	0.302	0.219	0.240	0.238	0.270	0.264	0.188	0.278	0.542	0.458	0.452	54.486
13	0.304	0.220	0.237	0.240	0.274	0.268	0.188	0.270	0.541	0.459	0.456	54.637
14	0.301	0.222	0.234	0.243	0.268	0.284	0.191	0.257	0.535	0.465	0.474	53.640
15	0.305	0.222	0.235	0.238	0.279	0.267	0.193	0.261	0.540	0.460	0.460	52.948
16	0.307	0.221	0.236	0.237	0.281	0.264	0.191	0.263	0.543	0.457	0.456	53.822
17	0.305	0.222	0.236	0.237	0.279	0.264	0.195	0.263	0.541	0.459	0.458	53.194
18	0.305	0.222	0.240	0.233	0.279	0.254	0.194	0.273	0.545	0.455	0.448	53.054
19	0.308	0.219	0.231	0.241	0.282	0.274	0.189	0.254	0.540	0.460	0.464	53.359
20	0.305	0.219	0.236	0.240	0.274	0.277	0.190	0.259	0.540	0.460	0.467	54.470
21	0.305	0.222	0.234	0.240	0.276	0.273	0.194	0.257	0.538	0.462	0.467	53.840
22	0.305	0.221	0.234	0.239	0.276	0.272	0.193	0.258	0.539	0.461	0.466	53.705
23	0.305	0.222	0.234	0.239	0.276	0.273	0.194	0.257	0.539	0.461	0.466	53.745
24	0.306	0.221	0.233	0.240	0.280	0.273	0.191	0.256	0.539	0.461	0.464	53.546
25	0.305	0.222	0.232	0.241	0.276	0.273	0.193	0.257	0.537	0.463	0.466	53.349
26	0.303	0.223	0.233	0.241	0.273	0.275	0.196	0.256	0.535	0.465	0.471	53.914
27	0.303	0.223	0.232	0.241	0.273	0.275	0.196	0.256	0.535	0.465	0.471	53.800
28	0.302	0.224	0.231	0.243	0.273	0.279	0.196	0.252	0.533	0.467	0.475	54.002
29	0.304	0.222	0.233	0.241	0.275	0.274	0.194	0.257	0.536	0.464	0.468	53.752
30	0.303	0.224	0.231	0.242	0.273	0.278	0.196	0.253	0.534	0.466	0.474	53.895
31	0.303	0.223	0.233	0.241	0.274	0.274	0.195	0.258	0.536	0.464	0.469	53.803
32	0.303	0.220	0.234	0.244	0.273	0.281	0.180	0.265	0.537	0.463	0.462	53.837
33	0.304	0.220	0.237	0.239	0.280	0.273	0.180	0.267	0.541	0.459	0.453	53.339
34	0.303	0.221	0.237	0.238	0.276	0.269	0.183	0.272	0.541	0.459	0.452	54.287
35	0.298	0.222	0.235	0.245	0.260	0.284	0.184	0.271	0.534	0.466	0.469	53.712
36	0.297	0.222	0.238	0.242	0.274	0.272	0.181	0.273	0.535	0.465	0.453	55.105
37	0.302	0.221	0.236	0.240	0.271	0.276	0.184	0.269	0.539	0.461	0.460	54.092
38	0.302	0.222	0.235	0.241	0.270	0.276	0.187	0.266	0.537	0.463	0.464	54.774
39	0.305	0.220	0.236	0.240	0.280	0.272	0.178	0.269	0.541	0.459	0.450	54.418
40	0.304	0.222	0.237	0.237	0.274	0.266	0.191	0.270	0.541	0.459	0.457	53.926
41	0.304	0.221	0.237	0.239	0.277	0.267	0.185	0.272	0.540	0.460	0.452	54.478
42	0.303	0.221	0.237	0.239	0.276	0.268	0.184	0.273	0.541	0.459	0.452	54.450
43	0.304	0.220	0.237	0.239	0.276	0.268	0.184	0.272	0.541	0.459	0.453	54.463
44	0.306	0.219	0.236	0.239	0.280	0.271	0.181	0.268	0.542	0.458	0.452	52.838
45	0.300	0.224	0.235	0.241	0.270	0.274	0.194	0.263	0.535	0.465	0.467	52.609
46	0.303	0.221	0.238	0.238	0.272	0.267	0.190	0.271	0.541	0.459	0.457	52.735
47	0.301	0.222	0.237	0.240	0.270	0.271	0.191	0.268	0.538	0.462	0.462	54.245
**48**	0.303	0.223	0.232	0.242	0.278	0.281	0.192	0.248	0.535	0.465	0.474	53.968

### Compositional properties of ORFs of 48 polioviruses genomes

**Figure 1:**
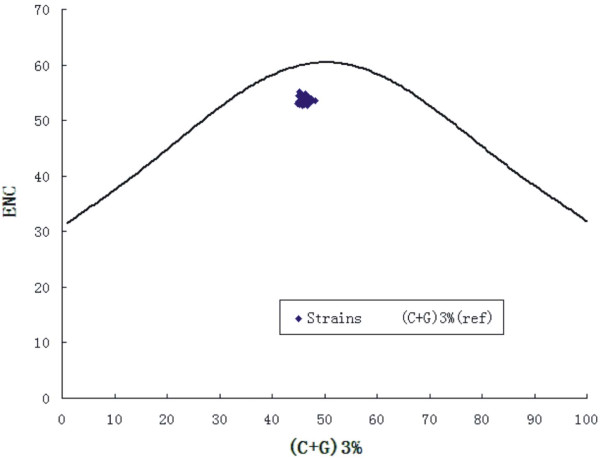
**Graphs showing the relationship between the effective number of codons (ENC) and the GC content of the third codon position (GC_3_)**. The curve indicates the expected codon usage if GC compositional constraints alone account for codon usage bias.

The values of A, U, C, G and C+G were compared with the values of A_3_, C_3_, G_3_, U_3_, (G+C) _3_, respectively. An interesting and complex correlation was observed. In detail, the (C+G)_3 _have highly significant correlations with A, U, C, G and C+G, respectively, indicating C+G may reflect interaction between mutation pressure and natural selection. However, the A have no correlation with A_3_, G_3 _and C_3_, and U have no correlation with A_3 _(Table 4). Both cases suggested that the nucleotide constraint possibly influence synonymous codon usage of polioviruses. In addition, the correlation between the Axis 1 (calculated by PCA) and the values of A, C, G, U, A_3_, C_3_, G_3_, U_3_, (G+C), (G+C)_3 _of each strain was also analyzed. The significant correlation was found between nucleotide compositions and synonymous codon usage to some extent excluding Axis 1 and the value of A (Table 4). The analysis revealed that most of the codon usage bias among ORFs of polioviruses strains was directly related to the base composition. Finally, the ENC-plot [ENC plotted against (G+C)_3_%] was used as a part of general strategy to investigate patterns of synonymous codon usage and all of the spots lie below the expected curve (Figure 1). These imply that the codon bias can be explained mainly by an uneven base composition, in other words, by mutation pressure rather than natural selection.

**Table 4:** Correlation analysis between the A, U, C, G contents and A _3_, U _3_, C _3_, G _3 _contents in ORF of 48 polioviruses genomes

	*A_3_*	*U_3_*	*G_3_*	*C_3_*	*(G+C) 3*	Axis 1
**A**	*r *= -0.093^N^	*r *= -0.303*	*r *= -0.169^N^	*r *= -0.185^N^	*r *= -0.287*	*r *= -0.126
**U**	*r *= -0.078^N^	*r *= 0.905**	*r *= -0.422**	*r *= -0.573**	*r *= -0.706**	*r *= -0.782**
**G**	*r *= -0.285*	*r *= -0.341*	*r *= 0.641**	*r *= 0.195^N^	*r *= 0.777**	*r *= 0.556**
**C**	*r *= -0.529**	*r *= -0.509**	*r *= -0.014^N^	*r *= 0.913**	*r *= 0.461**	*r *= 0.466**
G+C	*r *= -0.544**	*r *= -0.599**	*r *= 0.307 *	*r *= 0.807**	*r *= -0.851**	*r *= 0.708**
**Axis 1**	*r *= 0.541**	*r *= -0.700**	*r *= 0.360*	*r *= 0.401**	*r *= 0.502**	

Note: ** Means p < 0.01

* Means 0.01 < p < 0.05

^N ^Means no correlation

### Effect of other potential factors on codon usage

Principal component analysis was carried out to identify the codon usage bias among ORFs. From which we could detect one major trend in the Axis 1 which accounted for 20.815% of the total variation, and another major trend in the Axis 2 for 16.273% of the total variation. A plot of the Axis 1 and the Axis 2 of each gene was shown in Additional file 1, Figure S1. Obviously, those polioviruses belong to the same genotype tends to come together (except strain 48, isolated from Finland). Compared with the scattered groups of polioviruses genotype 1, genotype 2 and 3 strains aggregated more tightly to some degree. Although this graph is a little complex, it seems that there is a clear geographical demarcation in the polioviruses genotype 1 such as the VDPV strains isolated from USA, Dominica, China mainland and Taiwan. These may indicate that geographic is another factor on codon usage bias.

**Figure 2:**
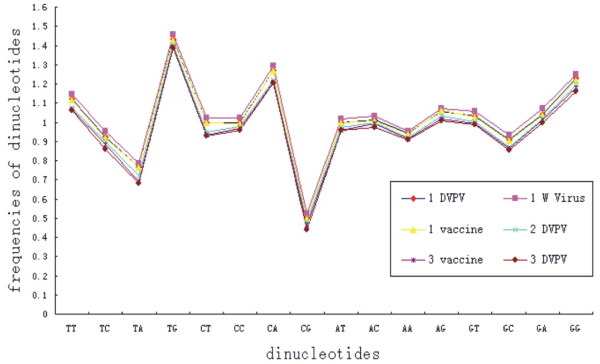
**Comparison the relative dinucleotide abundance in polioviruses DVPVs genotype 1, live attenuated virus genotype 1, wild viruses genotype 1, DVPVs genotype 2, DVPVs genotype 3 and live attenuated virus genotype 3**.

The frequencies of occurrence for dinucleotides were not randomly distributed and no dinucleotides were present at the expected frequencies. And the frequency of CpG and TpA was significantly low at all codon positions for coding region of 48 polioviruses genomes (mean ± S.D. = 0.490 ± 0.012; and mean ± S.D. = 0.748 ± 0.034. both < 0.78). The relative abundance of CpA and TpG also showed slight deviation from the ''normal range'' (mean ± S.D. = 1.253 ± 0.032 and 1.423 ± 0.023, respectively) (Table 5). In addition, the RSCU values of the eight codons containing CpG (CCG, GCG, UCG, ACG, CGC, CGG, CGU, and CGA) were analyzed, to reveal the possible effects of CpG under-represented on codon usage bias. All of these eight codons were not preferential codons and were markedly suppressed. The six codons containing TpA (UUA, CUA, GUA, UAU, UAC and AUA) were suppressed too. Conversely, the RSCU values of the eight codons containing CpA (UCA, CCA, ACA, GCA, CAA, CAG, CAU, CAC) and five codons containing UpG (UUG, GUG, UGU, UGC, CUG) are high, and most of them (8 out of 13) were preferential codons (Table 2 and Table 5). In addition, compared with DVPVs and live attenuated strain of polioviruses genotype 1, the wild viruses has higher frequencies of dinucleotides including CpG (Figure 2 and Table 5).

**Table 5:** Relative abundance of the 16 dinucleotides in ORF of 48 polioviruses

*Dinucleotides*	*Range^a^*	***Mean ± S.D***^*b*^	*DVPV 1*	*Wild viruses*	Vaccine
ApA	0.909-0.965	0.935 ± 0.016	0.943	0.955	0.937
ApG	0.993-1.083	1.048 ± 0.019	1.058	1.073	1.060
ApT	0.955-1.041	0.989 ± 0.019	0.999	1.018	0.999
ApC	0.960-1.040	1.007 ± 0.016	1.013	1.030	1.010
GpA	0.987-1.094	1.035 ± 0.020	1.043	1.071	1.038
GpG	1.140-1.266	1.215 ± 0.024	1.228	**1.249**	1.221
GpT	0.986-1.079	1.023 ± 0.021	1.033	1.057	1.028
GpC	0.846-0.950	0.895 ± 0.028	1.228	**1.249**	1.221
CpA	**1.204-1.308**	**1.253 ± 0.032**	**1.270**	**1.293**	**1.268**
CpG	**0.418-0.538**	**0.490 ± 0.012**	**0.499**	**0.522**	**0.496**
CpT	0.920-1.039	0.979 ± 0.033	0.995	1.022	0.997
CpC	0.952-1.035	0.991 ± 0.012	0.999	1.021	0.995
TpA	**0.669-0.801**	**0.748 ± 0.034**	**0.766**	0.787	**0.766**
TpG	**1.386-1.474**	**1.423 ± 0.023**	**1.434**	**1.457**	**1.427**
TpT	1.059-1.164	1.106 ± 0.029	1.122	1.144	1.118
**TpC**	0.824-0.973	0.914 ± 0.026	0.925	0.953	0.916

Note: The boldface means that the dinucleotide was over-represented or under-represented.

^a ^The range of coding region of 48 polioviruses's relative dinucleotide ratios

^b ^Mean values of coding region of 48 polioviruses's relative dinucleotide ratios  ±  S.D

Furthermore, we also performed a linear regression analysis on ENC value and gene length of ORFs of 48 polioviruses genomes. However, there was no significant correlation between codon usage and gene length in these virus genes (Spearman *P *> 0.05).

## Discussion

Studies of synonymous codon usage in viruses can reveal much about viral genomes [25]. The overall codon usage among 48 ORFs of polioviruses was analyzed in this study. First, the ENC values of all the poliovirus samples were analyzed, and the results showed that the majority of polioviruses do not have a strong codon bias (mean ENC = 53.754 > 40). In addition, together with published data on codon usage bias among some RNA viruses, such as BVDV, H5N1 influenza virus and SARS-covs with mean values of 51.43, 50.91 and 48.99, respectively, one possible explanation for this is that the weak codon bias of RNA virus is advantageous to replicate efficiently in vertebrate host cells, with potentially distinct codon preferences [26-28].

Natural selection and mutation pressure are thought to be the main factors that account for codon usage variation in different organisms [29-31]. In this study, the general association between codon usage bias and base composition suggests that mutational pressure, rather than natural selection is the mainly factors on codon usage pattern of polioviruses.

Codon usage can also be strongly influenced by underlying biases in dinucleotide frequency, which differs greatly among organisms. Specifically, after accounting for dinucleotide biases, the proportion of codon usage bias explained by mutation pressure often increases, as seen in human RNA viruses [25]. Our study revealed that CpG and the eight CpG-containing codons are notably deficient in ORFs of 48 poliovirus genomes. The explanation for the CpG deficiency is immunologic escape. A high CpG content may be detrimental to small DNA (or RNA) viruses, as unmethylated CpGs are recognized by the host's innate immune system (Toll-like receptor 9) as a pathogen signature [32]. As with vertebrate genomes, methylated viral genomes would face a high chance of mutation at CpGs, that would result in a reduction of this dinucleotide [9,33]. We found that DVPVs and live attenuated virus of genotype 1 have lower frequencies of CpG dinucleotide compare with wild viruses of polioviruses genotype 1. The most popular explanation for lower frequencies of CpG in ORFs of DVPV genomes is that when OPV viruses turning into VDPV genotype 1, a lower frequencies of CpG dinucleotide maybe help VDPV out of the host immunity.

Although it seems speculative and complex, some researchers have found that reduction of the rate of poliovirus protein synthesis through large-scale utilization of codons that are not optimal has caused attenuation of viral virulence by lowering specific infectivity [34]. Therefore, the information from this study may not only have theoretical value in understanding poliovirus evolution (especially for DVPVs genotype 1), but also have practical value for the development the poliovirus vaccine. However, a more comprehensive analysis is needed to reveal more information about codon usage bias variation within poliovirus and other responsible factors.

## Conclusions

The information from this study may not only help to understand the evolution of the poliovirus, especially for DVPVs genotype 1, but also have potential value for the development of poliovirus vaccines.

## Competing interests

The authors declare that they have no competing interests.

## Authors' contributions

JZ, MW and YL designed the study and drafted the manuscript. WL, JZ, HC and LM collected the data and participated in the sequence alignment. YD and YG performed the statistical analysis. All authors read and approved the final manuscript.

## Supplementary Material

Additional files 1 **Figure S1**. A plot of the values of the Axis1a (20.82%) and the Axis2a (16.27%) of each ORF in principle component analysis. Click here for file
