# Supplementary figures and images for: Analysis of codon usage and nucleotide composition bias in polioviruses
Source: Virol J. 2011 Mar 30;8:146. doi: 10.1186/1743-422X-8-146 (PMC3079669; doi:10.1186/1743-422X-8-146)

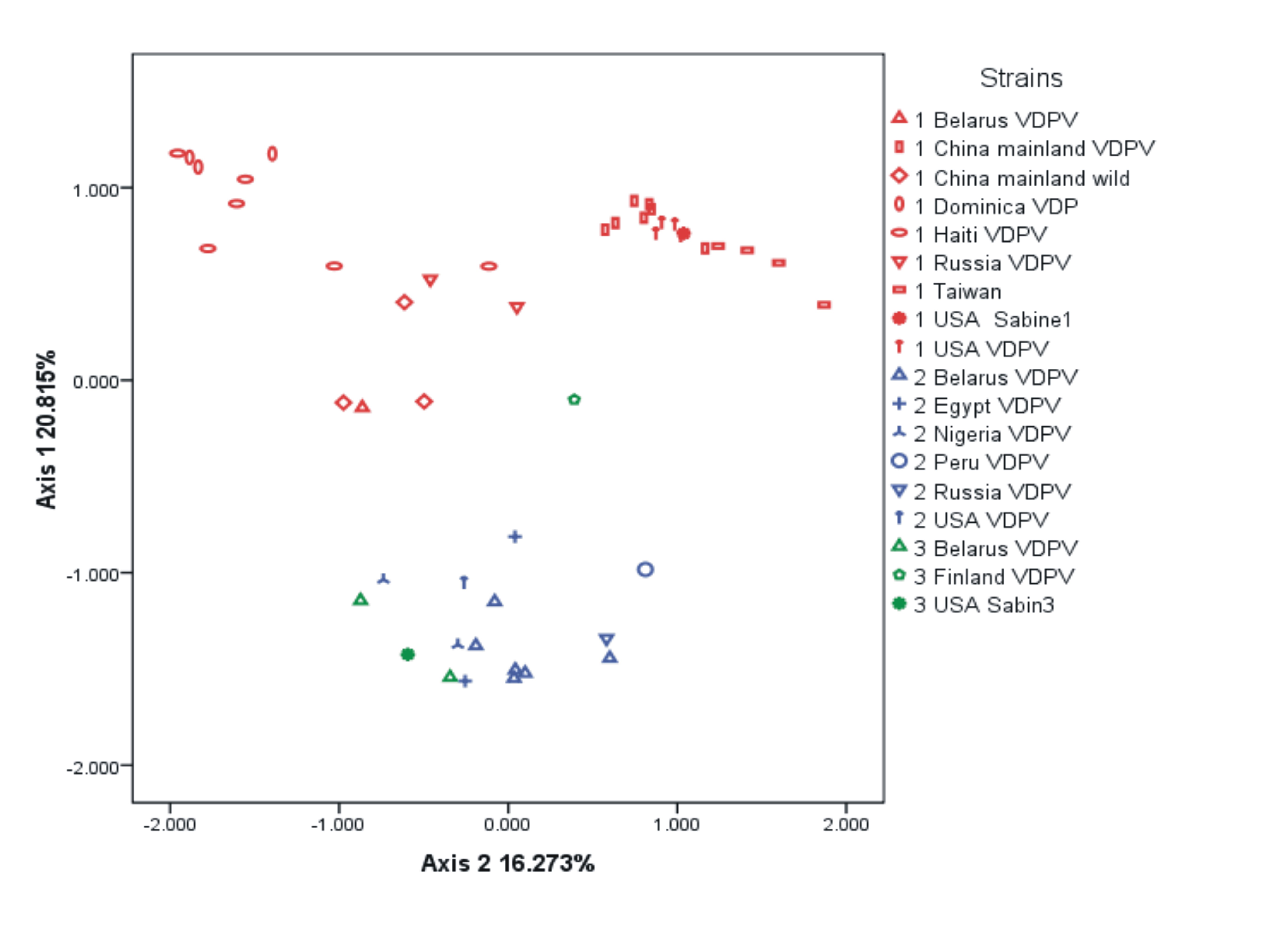

Supplement: Additional files 1 — Figure S1. A plot of the values of the Axis1a (20.82%) and the Axis2a (16.27%) of each ORF in principle component analysis. [file 1743-422X-8-146-S1.JPEG]
